# Chronic Hospitalized and Household Maintained Consumers; Characteristics and Differences Among Referees to the Iranian Society Supporting Individuals With Schizophrenia

**Published:** 2014

**Authors:** Nader Mansouri, Seyed Kazem Malakouti, Narges Chimeh, Shirin Rezvanifar, Mostfa Mohseni, Elham Mansouri

**Affiliations:** 1 Psychologist, Family Research Institute, Shahid Beheshti University, Tehran, Iran.; 2 Professor, Department of Psychiatry, Iran University of Medical Sciences, Mental Health Research Centre, Tehran, Iran.; 3 Professor, Family Research Institute, Shahid Beheshti University, Tehran, Iran.; 4 Psychologist, Department of Psychology, School of Psychology and Educational Sciences, Allameh Tabatabee University, Tehran, Iran.; 5 Psychologist, Family Research Institute, Shahid Beheshti University, Tehran, Iran.; 6 Psychologist, Department of Psychology, School of Psychology and Educational Sciences, Shahid Beheshti University ,Tehran, Iran

**Keywords:** Consumer, Differences, Household Maintenance, Psychiatric Hospitalization, Schizophrenia

## Abstract

**Objective:** Providing treatment and educational services for consumers and their caregivers is more effective if those services are implemented based on their characteristics and differences. To partly address this objective, the present study aimed to describe and compare characteristics and differences of chronic hospitalized and household maintained consumers and their caregivers who were regular users of educational and rehabilitation programs of the Iranian Society Supporting Individuals with Schizophrenia (ISSIS) in Tehran, Iran.

**Methods:** Two hundred and thirty one consumers were evaluated based on demographics, and clinical and symptom-related characteristics. Their caregivers (n = 231) were independently evaluated based on their knowledge on schizophrenia, family function, burden, and availability of social services and support for them. Data were analyzed by performing independent sample t-test and Mann-Whitney U.

**Results: **The study findings revealed hospitalized consumers were older, had longer length of illness, greater severity of positive and negative symptoms and lower efficacy in basic life skills in comparison with household maintained consumers. The caregivers of the hospitalized consumers had greater objective and subjective burdens and lower knowledge on schizophrenia in comparison with caregivers of household maintained consumers. While household maintained consumers had more access to medical insurance, their caregivers had more access to the supportive organizations, more availability of substitute caregiver and assistant caregiver compared with caregivers of hospitalized consumers.

**Conclusion:** Our findings suggest the need to promote specific interventions and treatment programs for Iranian consumers and their caregivers based on their characteristics and differences due to schizophrenia.

**Declaration of interest:** None.

## Introduction

Schizophrenia is a chronic mental illness. Some studies show up to 90% of individuals with mental disorders live with relatives who provide long-term practical and emotional support for them (1, 2) and caregivers’ burden increases with more contact with patient and the time patients live with their families (3). Family caregivers engaged in providing care for a patient member may experience grief for loss of patient member’s former personality and family lifestyle (4).

Family burden in caring patient members with long-term disorder may lead to objective and subjective difficulties (5) and need educational and treatment interventions. Caregivers may experience objective burden such as facing disruption of family relationships, constrained levels of social, work and leisure activities and financial problems. They may experience subjective burden such as feeling of loss, anxiety, sadness and pressure in confronting with disturbing behaviors of their patient family members and frustration that change in relationships impose on them (6). 

In Iran, caregivers play a significant role in supporting family members with schizophrenia. Some studies show Iranian caregivers of patients with schizophrenia endure significant burden in maintaining their patient members (7), especially when psychopathology of the patients and deficiencies in basic life skills are manifested among patient members (8). A recent study showed Iranian caregivers experience a significant level of anxiety, depression, disturbance in sleep, and psychosomatic problems and 76% of the parents reported disturbance in their mental health status (9) but despite all these problems, Iranian caregivers prefer to maintain their patient members and they reported need for hospital beds at the time of relapse and prefer rehabilitation and educational programs face schizophrenia (10). In recent years, some sporadic studies have shown case management (11) and family interventions (12) have been effective in both reducing burden of care giving among Iranian caregivers and some symptoms of the patients but such interventions can be more effective if they are implemented based on characteristics and differences of main groups of consumers and their caregivers.

In regard with critical limitations in financial resources for treating Iranian consumers, the necessity for implementing treatment services for consumers and educational services for their caregivers with optimal effectiveness is a health priority in Iran. This trend is budget saving and may contribute to maximum treatment outcome but this goal is achieved when characteristics and differences of the two mentioned groups of consumers are identified and treatment and educational programs are implemented based on these identified characteristics and differences. This issue may also contribute to promote the efficacy and effectiveness of the implemented services.

To partly contribute to this aim, the current study was designed to describe and compare demographics, clinical and symptom-related characteristics among the two mentioned groups of consumers and knowledge on schizophrenia, family function, burden, and availability of social services and support among their caregivers referring to the Iranian Society Supporting Individuals with Schizophrenia (ISSIS) as one of major centers for consumers and their caregivers in Tehran, Iran.

## Materials and Methods

***Participants***

Two hundred and thirty one consumers with their main caregivers from (ISSIS) were eligible to enter into the study and agreed to be interviewed and completed all the measurements. Of the 231 consumers, 106 consumers were current chronically hospitalized (group 1) and 125 of them were current household maintained (group 2). 

Those consumers who were diagnosed as mentally retarded, or were diagnosed with comorbid psychiatric and/or severe physical disorder and current use of substance and alcohol were excluded. 

Current chronically hospitalized consumers were characterized by at least one time of hospitalization in psychiatric hospitals in each year of the past 5 years because of severe presence of psychiatric symptoms and more than 5 years of psychiatric diagnosis with schizophrenia while current household maintained consumers were characterized by constant residence with families with or without history of psychiatric hospitalization in the past 5 years and less than 5 years of psychiatric diagnosis with schizophrenia.

The majority of the consumers were elderly single and unemployed males but we also recruited female consumers while the majority of caregivers were elderly mothers who were homemakers with limited education and financial resources but we also interviewed spouses, fathers, brothers and other family members of the consumers. All the participants were provided with oral and written informed consent forms and the protocol of the study was approved by the institutional review board of Shahid Beheshti University in Tehran.

***Assessment procedure***

*Patient-related assessment: *In the first phase, diagnosis of schizophrenia in participants was confirmed by using the Structured Clinical Interview for the Diagnostic and Statistical Manual of Mental Disorders 4^th^ edition (DSM-IV), Psychotic Disorders Version, and severe combined immune deficiency (SCID-I) (13). Demographic and clinical characteristics of the participants were completed based on a questionnaire designed to do so. The severity of positive symptoms of schizophrenia was measured by the Scale of Assessment of Positive Symptoms (SAPS) (14) and the severity of negative symptoms was measured by the Scale of Assessment of Negative Symptoms (SANS) (15). In this study, internal reliabilities via Cronbach’s alpha coefficients for positive and negative symptoms were 0.83% and 0.87%, respectively. 

Kohlman Evaluation of Living Skills (KELS) (16) was administered to measure abilities of participants in basic life skills. In this study, the internal reliability via Cronbach’s alpha coefficient was obtained 0.72%. 

*Caregiver-related assessment: *After completing socio-demographics for caregivers, Knowledge Questionnaire for Caregivers was administered to evaluate the family knowledge about schizophrenia. A brief version of this questionnaire developed by Khazailie with 31 questions was administered covering the symptoms, treatment and family’s awareness and behavior toward the patient (17). The reliability of this questionnaire within a week was 0.82%. Cronbach’s alpha coefficient in the present study was 0.75%.

Family Experience Interview Schedule (FEIS) was administered to evaluate family characteristics and burden (18). In this study, a short version of FEIS including 41 questions was administered and internal reliability via Cronbach’s alpha coefficient within a week was 0.89% (11). In this study, Cronbach’s alpha coefficient was 0.90%.

To evaluate the perceived social support, Social Support Scale (SSS) (19) was administered. Internal reliability via Cronbach’s alpha coefficient of this scale in the present study was 0.70%.

To evaluate family function, we administered Family Assessment Device (FAD) devised by Epstein et al. In the original study, internal Cronbach’s alpha ranged from 0.72-0.92% between the subscales (20). In this study, Cronbach’s alpha ranged between 0.61-0.86%, respectively.

***Data Analysis***

A series of independent sample t-test and Mann-Whitney U analysis tests in SPSS for Windows 16.0 (SPSS Inc., Chicago, IL, USA) performed to explore the differences between the two groups.

## Results

***General characteristics of the patients***

The age range of the study subjects was 18 to 64 years and the majority of them were males. Mean age of the group 1 (68.1 ± 40.9 years) was similar to the mean age of group 2 (67.9 ± 35.8 years). Moreover, the number of lifetime psychiatric hospitalization in group1 was (5.5 ± 9.7) times that was dramatically more than the similar number (2.3 ± 3.2) for group 2. Length of illness in the group 1 was 9.4 ± 18.2 years that was considerably more than length of illness e.g. 3.8 years in group 2 (Table 1).

The caregivers’ age range was 19 to 84 years. The majority of the caregivers were elderly women. A considerable number of caregivers in group 1 were mothers (32.1%), sisters (19.8%), fathers and brothers with the same proportion (17.9%); while the proportion of mothers (52.8%), fathers (18.4%) and spouses (16.0%) in caring household maintained subjects were more reported while the remaining care givers were other family members(Table 2).

*Characteristics related to the consumers*

The hospitalized subjects were older (t = 3.72; p *< *0.002), had longer length of illness (t = 3.69; p *< *0.003) (Table 3), greater severity of negative symptoms (t = 8.12; p *<* 0.001), and positive symptoms (t = 2.045; p *<* 0.042) and lower efficacy in basic life skills (t = 8.48; p *<* 0.001) compared with household maintained consumers (Table 4).

*Characteristics related to the caregivers*

Caregivers of the hospitalized subjects reported experiencing greater objective burden (t = 4.24; p < 0.001), subjective burden (t = 4.70; p < 0.003) and less knowledge on schizophrenia (t= -5.78; p < 0.001) compared with caregivers of household maintained consumers (Table 5).

*Availability of supportive and health-related services*

Caregivers of the household maintained subjects reported availability of more social support (t = -8.98; p < 0.01) (Table 6), more availability of medical insurance (t = -2.07; p *< *0.038), more access to supportive organization (t = -3.76; p *< *0.001) and availability of assistant caregiver (t = -3.60; p *< *0.001) and substitute caregiver (t = -2.33; p *< *0.020) compared with caregivers of the hospitalized subjects (Table 7).

**Table 1:** Demographics of the consumers

**Variable**	**Characteristics **	**Group 1 (n = 106)**	**Group 2 (n = 125)**
**Gender**	Male	81 (76.4%)	102 (81.6%)
	Female	25 (23.6%)	23 (18.4%)
**Mean age (Years)**		68.1 ± 40.9	67.9 ± 35.8
**Age range (Years)**		18-64	22-60
**Marital status**	Never married	65 (61.3%)	90 (72%)
	Currently married	13 (12.3%)	24 (19.2%)
	Divorced	26 (24.5%)	9 (7.2%)
	Widower / widow	2 (1.9%)	2 (1.6%)
**Education (Years)**	Less than 12 years	63 (59.4%)	46 (36.8%)
	12 years	31 (29.2%)	64 (51.2%)
	More than 12 years	12 (11.2%)	15 (12.0%)
**Job status**	Yes	11 (10.4%)	27 (21.6%)
	No	95 (89.6%)	98 (78.4%)
**Medical insurance**	Yes	33 (31.1%)	108 (86.4%)
	No	73 (58.9%)	17 (13.6%)
**Mean of lifetime psychiatric hospitalization**		05.5 ± 9.7	02.3 ± 3.2
**Frequency of lifetime psychiatric hospitalization**		01-25	00-10
**Length of illness (Years)**		9.4 ± 18.20	3.8 ± 14.7000

**Table 2:** Demographic characteristics of the caregivers

**Variable**	**Characteristics**	**Group 1 (n = 106)**	**Group 2 (n = 125)**
**Gender**	Male	40 (37.7%)	25 (20%)
	Female	66 (62.3%)	100 (80%)
**Mean age (Years)**		54.1 ± 14.3	55.0 ± 11.7
**Age range (Years)**		19-80	25-84
**Marital status**	Currently married	61 (57.5%)	93 (74.4%)
	Widower / widow	21 (19.8%)	22 (17.6%)
	Divorced	15 (14.2%)	4 (3.2%)
	Never married	9 (8.5%)	6 (4.8%)
**Education (Years)**	Less than 12	59 (34.2%)	56 (46.5%)
	12	29 (27.3%)	58 (46.4%)
	More than 12	18 (17%)	11 (8.8%)
**Income (Per month)**	Less than $300	48 (45.3%)	54 (43.2%)
	USD 300-750	53 (50%)	66 (54.5%)
	More than $750	5 (4.7%)	5 (4%)
**Type of relation with consumers**	Mother	34 (32.1%)	66 (52.8%)
	Father	19 (17.9%)	23 (18.4%)
	Spouse	8 (7.5%)	20 (16.0%)
	Sister	21 (19.8%)	12 (9.6%)
	Brother	19 (17.9%)	2 (1.6%)
	Child	3 (2.8%)	1 (8%)
	Grandmother	2 (1.9%)	1 (8%)

**Table 3:** Subject differences in related demographic and clinical variables

**Variable**	**Group**	**Mean (SD)**	**t-test**	**df**	**P-value**
**Age**	1	41.03 (11.02)	-3.72	229	0.002*
	2	35.93 (9.81)			
**Length of illness (Years)**	1	18.29 (9.04)	-3.69	229	0.003*
	2	14.07 (8.31)			
**Number of lifetime Hospitalization**	1	09.76 (5.85)	-0.210	225	0.834*
	2	11.61 (90.53)			

* p < 0.01

**Table 4:** Subjects differences in symptom-related and basic life skills variables

**Variable**	**Group**	**Mean (±SD)**	**t-test**	**df**	**P-value**
**Negative symptoms**	1	66.13 (±12.35)	8.120	192	0.001**
	2	53.17 (±9.74)			
**Positive symptoms**	1	63.06 (±12.54)	2.045	192	0.042**
	2	59.75 (±9.83)			
**Basic life skills**	1	8.06 (±2.00)	8.480	192	0.001**
	2	5.72 (±1.83)			

* p < 0.05;

** p < 0.01

**Table 5:** Caregivers’ differences in family characteristics and burden variables

**Variable**	**Group**	**Mean (±SD)**	**t-test**	**df**	**P-value**
**Problem solving**	1	02.08 (±0.533)	-0.407	203	0.194*
	2	02.11 (±0.546)			
**Communications**	1	02.26 (±0.147)	1.210	203	0.680*
	2	02.19 (±0.408)			
**Roles**	1	02.61 (±0.462)	0.610	203	0.540*
	2	02.57 (±0.421)			
**Affective responsiveness**	1	02.31 (±0.505)	0.136	203	0.890*
	2	02.30 (±0.577)			
**Affective involvement**	1	02.39 (±0.503)	1.180	203	0.236*
	2	02.31 (±0.518)			
**Behavior control**	1	02.46 (±0.457)	1.510	203	0.249*
	2	02.39 (±0.428)			
**General functioning**	1	02.42 (±0.480)	0.160	203	0.870*
	2	02.41 (±0.533)			
**Objective burden**	1	26.97 (±5.85)	4.240	203	0.001*
	2	23.01 (±7.46)			
**Subjective burden**	1	71.47 (±13.63)	4.700	203	0.003*
	2	60.73 (±18.78)			
**Family knowledge**	1	20.33 (±3.18)	-5.780	203	0.001*
	2	22.77 (±2.84)			

* p < 0.01

**Table6:** Caregivers’ differences in the variable of perceived social support

**Variable**	**Group**	**Mean (±SD)**	**t-test**	**df**	**P-value**
**Social support**	1	5.24 (±2.59)	8.98	192	0.001*
	2	8.43 (±2.53)			

* p < 0.01

**Table7:** Subjects and caregivers’ difference in access to supportive and health-related services

**Variable**	**Group**	**Mean**	**Z score**	**P-value**
**Medical insurance**	1	106.20	-2.07	0.038*
	2	123.30		
**Supportive organization**	1	103.53	-3.76	0.001**
	2	130.70		
**Assistant caregiver**	1	098.89	-3.60	0.001**
	2	125.95		
**Caregiver substitute**	1	105.43	-2.33	0.020*
	2	123.10		

* p < 0.05;

** p < 0.01

## Discussion

In this study, we revealed some characteristics and differences between chronic hospitalized and household maintained consumers and their caregivers which met different importance. As a developing country with limited financial resources for treating consumers, identifying such characteristics and differences among the two main groups of consumers and their caregivers could reflect their treatment and educational needs and priorities which can be used in designing and implementing effective interventions.

We observed most of the consumers were male and the majority of their caregivers were females especially homemaker mothers. Our study findings are consistent with what we are observing in the developed countries that show patients with schizophrenia are typically male and their caregivers are female especially mothers (21, 22).

We found a greater proportion of elderly consumers with a longer length of illness in group 1 compared with group 2. Although these observations do not state causation but older age in group 1 could reflect longer years of challenge with schizophrenia disorder and long length of living of these patients with their disorder and its chronic severity. Elderly consumers were also likely not to be able to take care of themselves, and old age and prolonged duration of schizophrenia were likely to impose more subjective and objective burden on caregivers, and it was likely to result in greater inpatient admission of these patients.

This study finding was in consistent with studies in developed countries emphasizing older age of psychiatric patients as a burden (23) and was in contrast with some studies in developed countries that suggest shorter duration of illness is a burden for caregivers (24) not longer duration of illness which results in longer hospitalization.

We found that group 1 had lower efficacy in performing basic life skills and greater symptoms than group 2. This issue could reflect the chronic and disabling nature of schizophrenia disorder which disabled these patients in doing their daily routines. It may be also partly due to this issue that in family atmosphere, caregivers allocated more time and care to the basic needs of their disable members and showed more sympathy toward their affective needs which were likely reduce deficiency in basic life skills and severity of symptoms compared with crowded inpatient psychiatric wards of hospitals but whether chronic inpatient admission results in increasing deficiency in basic life skills and more severity of symptoms or these issues result in chronic psychiatric hospitalization among Iranian consumers is an avenue for future research. A study showed deficiency in carrying out life skills among consumers is associated with feeling burden among caregivers, and heavy costs (25). Moreover, some studies show clinical symptoms (26), the severity of patient symptoms (26) result in feeling burden by caregivers and patient’s dependence on caregiver. 

Caregivers of group 1 had more objective and subjective burden and lower level of knowledge on schizophrenia compared to the caregivers of group 2. Feeling burden and lower knowledge on schizophrenia were likely to result in feeling distress and burden and referring them to inpatient service. This study finding emphasizes family psycho-educational and supportive interventions that are considered a best practice in the treatment of schizophrenia (27).

We found a greater proportion of the caregivers of group 1 had more perceived social support and availability of medical insurance for their patients, more access to supportive organization, assistant caregiver and substitute caregiver during critical periods due to patient compared with caregivers of group 2. These issues were likely to reduce emotional and financial burden of care giving and encouraged the caregivers to continue caring of their patients at home and not to refer them to psychiatric hospitals.

These findings support other studies that show perceived social support by caregivers, availability of help from professional agencies, a larger number of social contacts, more emotional supporters and more social interaction an reduce burden and distress (26).

Our study had also several limitations. We limited our sample to ISSIS with mainly male consumers and a limited number of female consumers and elderly caregivers with limited education and income who agreed to cooperate with us which are subjects to further studies with larger sample populations and other caregivers such as employed caregivers and relatives but our sample was representative of the group we studied. In fact, we referred to many centers in Tehran for sampling; but they disagreed to cooperate with us. Survey studies may give a better picture of differences between chronic hospitalized and household maintained consumers which might be helpful in designing treatment programs based on their needs and differences. To sum up, our findings revealed a wide range of differences in domains of patient characteristics and family characteristics as well as availability of social and supportive services in a developing country like Iran. Interventions to support and develop the consumer’s ability to contribute to the household and management of their own care, such as skills training or wellness recovery, as well as promoting and developing educational programs for caregivers and access to social and supportive services are most likely to meet the needs and enhance the quality of life of people with mental illness and their families rather than inpatient service utilization. 
